# Photodynamic Effect of Ni Nanotubes on an HeLa Cell Line

**DOI:** 10.1371/journal.pone.0150295

**Published:** 2016-03-18

**Authors:** Muhammad Hammad Aziz, M. Fakhar-e-Alam, Mahvish Fatima, Fozia Shaheen, Seemab Iqbal, M. Atif, Muhammad Talha, Syed Mansoor Ali, Muhammad Afzal, Abdul Majid, Thamir Shelih Al.Harbi, Muhammad Ismail, Zhiming M. Wang, M. S. AlSalhi, Z. A. Alahmed

**Affiliations:** 1 Department of Physics, College of Science in Zulfi, Majmaah University, Zulfi, Saudi Arabia; 2 Department of Physics, COMSATS Institute of Information and Technology, Lahore, Pakistan; 3 Institute of Fundamental and Frontier Science, University of Electronic Science and Technology of China, 610054 Chengdu, China; 4 Department of Physics, GC University Faisalabad, Pakistan; 5 Department of Physics, The Islamia University of Bahawalpur, Bahawalpur, Pakistan; 6 Department of Physics, GC University Lahore, Lahore, Pakistan; 7 Department of Physics and Astronomy, College of Science, King Saud University, Riyadh, Saudi Arabia; 8 National Institute of Laser and Optronics, Nilore, Islamabad, Pakistan; 9 Deanship of Scientific Research, King Saud University, Riyadh 11421, Saudi Arabia; 10 Institute of Biomedical and Genetic Engineering (IBGE), 24 Mauve Area, G-9/1, Islamabad, Pakistan; Massachusetts General Hospital, UNITED STATES

## Abstract

Nickel nanomaterials are promising in the biomedical field, especially in cancer diagnostics and targeted therapy, due to their distinctive chemical and physical properties. In this experiment, the toxicity of nickel nanotubes (Ni NTs) were tested in an *in vitro* cervical cancer model (HeLa cell line) to optimize the parameters of photodynamic therapy (PDT) for their greatest effectiveness. Ni NTs were synthesized by electrodeposition. Morphological analysis and magnetic behavior were examined using a Scanning electron microscope (SEM), an energy dispersive X-ray analysis (EDAX) and a vibrating sample magnetometer (VSM) analysis. Phototoxic and cytotoxic effects of nanomaterials were studied using the Ni NTs alone as well as in conjugation with aminolevulinic acid (5-ALA); this was performed both in the dark and under laser exposure. Toxic effects on the HeLa cell model were evaluated by a neutral red assay (NRA) and by detection of intracellular reactive oxygen species (ROS) production. Furthermore, 10–200 nM of Ni NTs was prepared in solution form and applied to HeLa cells in 96-well plates. Maximum toxicity of Ni NTs complexed with 5-ALA was observed at 100 J/cm^2^ and 200 nM. Up to 65–68% loss in cell viability was observed. Statistical analysis was performed on the experimental results to confirm the worth and clarity of results, with p-values = 0.003 and 0.000, respectively. Current results pave the way for a more rational strategy to overcome the problem of drug bioavailability in nanoparticulate targeted cancer therapy, which plays a dynamic role in clinical practice.

## 1. Introduction

Astonishing, nanotechnology has moved the nano world with its magnificent physiochemical characteristics. It has affected a multitude of areas, including science, technology, energy production, aerospace, electronics, engineering, environmental remediation and medical health care. Currently, nickel nanomaterials have received the most attention in the health and medical fields because of their potential impact on the environment and human health. The ability of nanomaterials to penetrate basic biological structures, such as cellular organelles and cells, mostly depends on the suitable size of the nanomaterials [1–3]. The size, chemical composition and shape play vital roles in nanomaterial toxicity [2]. It has been reported that nanomaterials are more toxic than bulk materials [2–3]. Nickel nanomaterials have many unique properties, such as high stability and catalytic activity. However, their adverse effects on the environment and human health have also been investigated [4–7].

It has been reported that nickel nanowires (NWs) induce apoptotic cell death due to ROS generation and flow cytometric cell cycle arrest. Moreover, nanomaterials such as ferromagnetic nanowires induce tumor cell death; thus, they may have an application as an anticancer agent [8–9]. The internalization of nickel nanowires by osteoblastic osteosarcoma cells (UMR-106) and mouse osteoblastic cells (MC3T3-El) indicated that they could be used for variety of biological applications [9]. The International Agency of Research on Cancer has classified Ni compounds as carcinogenic to humans (group 1) and metallic Ni as possibly carcinogenic to humans (group 2B). ROS play a dynamic role in the apoptotic pathway [10], which leads to the oxidative stress that causes the destruction of mitochondria or DNA fragmentation. The toxicity of Ni-NPs is a result of oxidative stress from the release of excessive ROS (Ni-ions), which results in DNA damage as well as decreased intracellular glutathione (GSH) [7–9,11]. Internalization of Ni NWs can cause the loss of mitochondrial membrane potential and cell cycle arrest, as well as vascular shutdown, which may trigger ROS generation at the target site. This is key step for inducing apoptosis.

Many doped forms of nanotube composites, such as titanium dioxide NTs, have been synthesized by different techniques and used for several potential purposes [12–15]. Due to structure and size/shape versatility, much effort has been devoted to exploring the biocompatibility and toxicity of nanotubes. In this study, nickel nanotubes (Ni NTs) have a tendency to show toxic effects, which may lead to its potential role in cancer therapies.

The mortality rate of cervical cancer is 52%, with approximately 275,000 deaths reported in 2008, and 88% of cervical cancers cases registered in developing countries. Women, especially those of an advanced age, are the main target of this carcinoma. The HeLa cell line, is the first human cell line cultured over 5 decades of culturing research, and it is derived from an extremely aggressive cancer with poor prognosis, which means that radiation therapy alone is insufficient to eliminate the target cells [12]. The liberation of a significant amount of singlet oxygen and free radical production are the major factors for photodynamic reactions, which are key for treatment purposes. A limited preclinical study has been performed to establish potential advantages produced through the combination of nanomaterials, either alone or with 5-ALA and PDT [16–19]. Therefore, in our work, we have treated HeLa cell line with different doses of 5-ALA, Ni-NTs and a combination thereof to analyze the effectiveness with mediated PDT. Furthermore, by examining the cytotoxicity of Ni NTs, their potential role can be improved.

## 2. Materials and Methods

### 2.1 Preparation of nickel nanotubes

Template-assisted synthesis of preparing Ni NTs was discussed in our research approach. Ni-NTs were grown via electrodeposition in the nanopore pattern characteristic of anodic aluminum oxide (AAO). The optimization of purified aluminum foil used a 1.0 M solution of NaOH, followed by electrodeposition in ethanol solution. Anodization of aluminium foil was performed using 0.3 M H_2_C_2_O_4_ that was anodized at constant applied voltage. The electrodeposition cell contained three electrodes and salted metal electrolytes in fixed ratios of 0.2 g of NiSO_4_ and 6H_2_O as well as 2 g of H_3_BO_4_ in 100 ml distilled water. The positive ions of Ni moved to the working electrode, and Ni NTs were deposited along the walls of the template at the cathode. Next, the AAO template was dissolved in 1 M NaOH, followed by the collection of suspended Ni NTs from the solution via a magnet. Ni NTs were redistilled until a neutral pH was achieved.

### 2.2 Cell culturing

Cells were cultured in minimum essential medium (MEM) with approximately 10% Hank’s salts, 10% fetal bovine serum (FBS) and 2 mM of L-glutamine. Cells were kept in culture in an incubator at 37°C, with routine subculturing twice or thrice a week when approximately 75–85% cell confluency was attained. The 1D layer of cells was harvested via 0.20% trypsin [16–17].

### 2.3 Cytotoxicity and phototoxicity in HeLa cells

The harvested HeLa cells were resuspended in a fresh medium and seeded in 96-well microwell plates at the desired optical density of 1×10^5^cells/well. At the desired cell confluence, the culture medium was removed and the cells were washed twice with 100 μL of phosphate buffered saline (PBS). A stock solution of 5-ALA was prepared in PBS (pH 7.4), and it was kept in the dark due to its photosensitivity. Specifically serum free media was used for dilution to avoid secretion of porphyrin from the cells. Cells were incubated with serum-free MEM (200 μL) containing different concentrations of Ni-NTs and 5-ALA Ni NTs (40 nM, 80 nM, 120 nM, 160 nM and 200 nM) for 20 hours at 37°C [16–18]. Drug absorbance or uptake was assessed with a microplate reader measuring via optical density at 405 nm, a wavelength compatible with the photosensitizer used on the cells [20–21].

A laser source with a 630 nm wavelength was used to irradiate the clear bottom surface of cell culture plates. Radial confluency depends on the time of exposure of cells to the laser. As a control, some cells were not irradiated. Again, fresh growth medium was added back to the cultured cells after the end of radiation session, which was followed by incubation of the plate at 37°C for 24 hours.

### 2.4 Detection of reactive oxygen species (ROS) fluorescence

Intracellular ROS production was explored by applying the non-fluorescent dye carboxy-H_2_DCFDA (2,7-dichloro-dihydrofluorescein diacetate acetyl ester), which frequently interacts with the plasma membrane of HeLa cells exposed to Ni NTs. Once inside the cell, the acetate groups are hydrolyzed. The resulting compound is less prone to exiting the cell. This new compound is not fluorescent, but it may be capable of reacting with singlet oxygen as well as with free radicals and super oxide ions. This leads to a highly fluorescent species, as ROS will oxidize this new complex, resulting in a fluorescent product.

### 2.5. Cell viability assessment

Phototoxicity in cells was measured via the neutral red cell viability assay (NRA). This assay is based on calculating the mean proportion of cells that are alive by measuring the uptake of vital dye neutral red. The culture plates, refreshed with 50 mg/ml of this dye, were incubated for approximately 3 hours. Then, the media were removed and the cells rinsed with PBS.

Cellular viability was determined as a percentage by the following formula:

$$\%\;\text{cell viability}=\left({\text{Cells}}_{\text{treated}}–{\text{Cells}}_{\text{blank}}\right)/\left({\text{Cells}}_{\text{control}}–{\text{Cells}}_{\text{blank}}\right)\times 100$$

## 3. Results and Discussion

**Fig 1:**
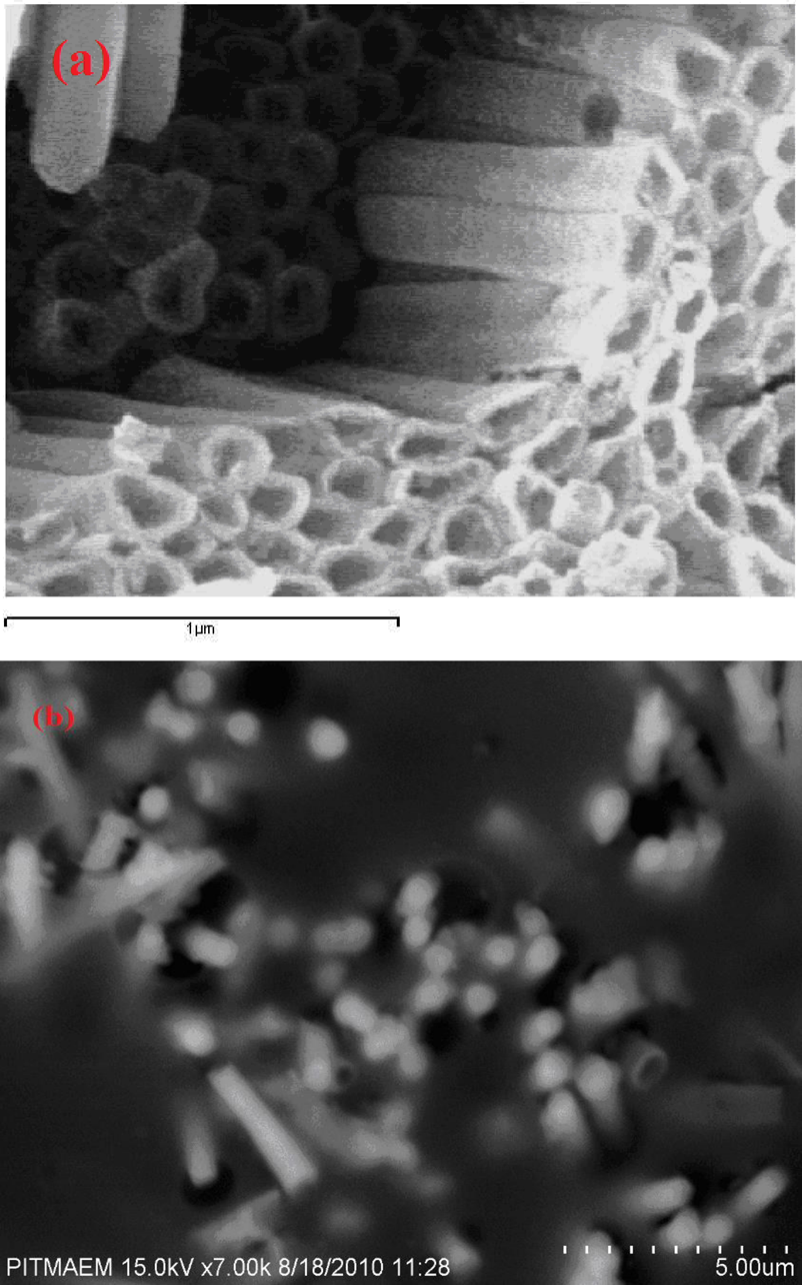
SEM of Nickel Nanotubes arrays (a) High magnification (b) low magnification. The morphology of the synthesized Ni nanotubes consists of single-walled nanotube arrays, as shown in the Fig 1(a,b). An SEM image of Ni nanotubes with inside diameters of approximately 200 nm and a wall thickness of approximately 25 nm is shown in (a). The lengths of Ni nanotubes are approximately 3.5 μm, as shown in (b).

The morphology of Ni NTs was observed using a JEOL JSM-6480 scanning electron microscope (SEM). The morphology of the synthesized Ni nanotubes consists of single-walled nanotube arrays, as shown in the Fig 1A and 1B. An SEM image of Ni nanotubes with inside diameters of approximately 200 nm and a wall thickness of approximately 25 nm is shown in Fig 1(A). The lengths of Ni nanotubes are approximately 3.5 μm, as shown in Fig 1(B).

**Fig 2:**
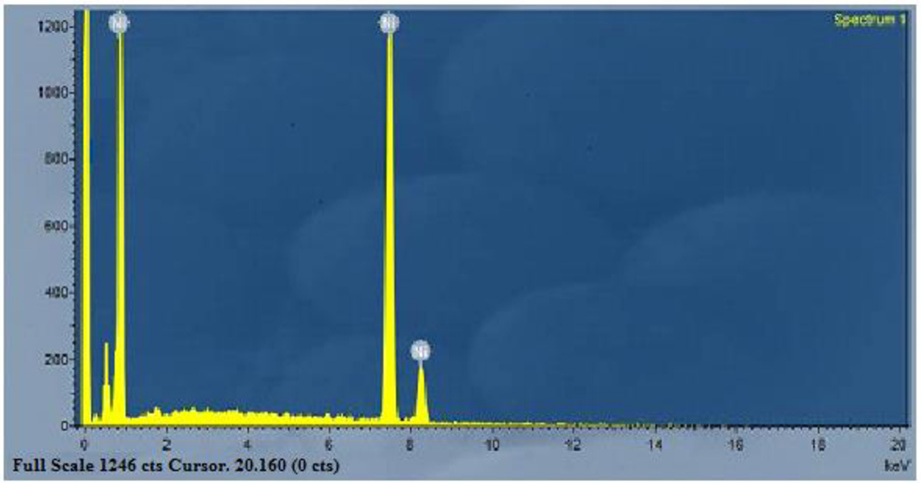
EDAX analysis of Ni nanotubes arrays. The EDAX readings prove that the required phase of Ni is present in the samples. Along with the Ni, some additional peaks are apparent, which is probably due to the presence of a substrate. EDAX indicates that the Ni nanotubes arrays were made of pure nickel.

The composition analysis of the Ni nanotubes arrays from the EDAX plot of the SEM images is shown in Fig 2. The EDAX readings prove that the required phase of Ni is present in the samples. Along with the Ni, some additional peaks are apparent, which is probably due to the presence of a substrate. EDAX indicates that the Ni nanotubes arrays were made of pure nickel.

**Fig 3:**
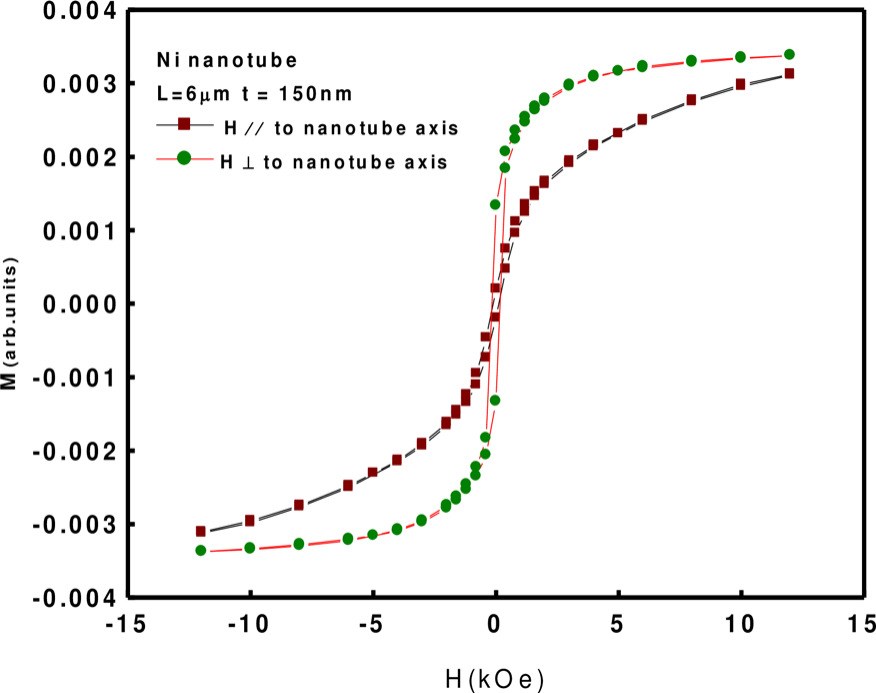
VSM OF Nickel Nanotubes. The in-plane and out-of-plane magnetic hysteresis loops at room temperature were investigated using vibrating sample magnetometer (VSM).

The in-plane and out-of-plane magnetic hysteresis loops at room temperature were investigated using vibrating sample magnetometer (VSM), as shown in Fig 3. The measured values from both loops are shown in Table 1.

**Table 1:** Measured values from both loops.

Magnetic Parameters	Magnetic Applied Field In Plane	Magnetic Applied Field Out of Plane
H_c_	118.83 O_e_	168.93 O_e_
M_s_	0.0031 emu	0.0035 emu

H_c_ = coercivity (O_e_), and M_s_ = saturation magnetization (emu). It is clear from Fig 2 that the easy axis is perpendicular to Ni Nanotubes, which is comparable to reports from literature [22]. The ferromagnetic behavior has been enhanced compared to bulk Ni [23].

**Fig 4:**
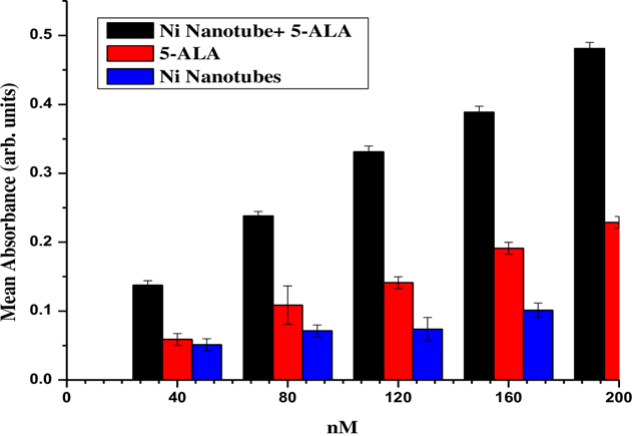
Absorbance of Ni Nanotubes, 5-ALA and complex of Ni Nanotubes with 5-ALA. Significant difference of bioavailability in HeLa cells was observed among Ni NTs, 5-ALA and the complex of Ni NTs and 5-ALA.

The current study suggests the importance of nickel nanotubes (Ni NTs) for cancer treatment, especially in a cervical cancer model. Fig 4 shows the cellular uptake of nickel nanotubes (Ni NTs), both individually and complexed with 5-ALA, in an HeLa cell line for 24 hours. J. Panchompoo et al. used electro-analytical detection of nickel/carbon nanotube (Ni/CNT) composites [24]. Significant difference of bioavailability in HeLa cells was observed among Ni NTs, 5-ALA and the complex of Ni NTs and 5-ALA, as depicted in Fig 4. Maximum accumulation of Ni NTs was recorded when 200 nM of the complex of Ni NTs and 5-ALA was applied to irradiated HeLa cells. The most prominent results were obtained in the case of 5-ALA compared to Ni NTs alone. Data were collected from experiments conducted in triplicate. In previously reported data, the anticancer effects of some nanomaterials, e.g., MnO_2_, ZnO, and Fe_2_O_3,_ were tested for feasibility by cytotoxic and phototoxic effects, but no significant results were obtained. Basically, the loss in malignant cell viability depends on the significant bioavailability of nanomaterials individually or complexed with photosensitizers, which can cause detectable oxidative stress and/or liberation of free radicals in a stained biological model and which have the ability to induce necrosis/apoptosis [17]. In this experiment, the optical density is a good measure of accumulation of Ni NTs+5-ALA in HeLa cells. In addition, a photosensitizer uptake as well as a loss of mitochondrial membrane potential depends upon the type of cell/biological model [17, 25].

**Fig 5:**
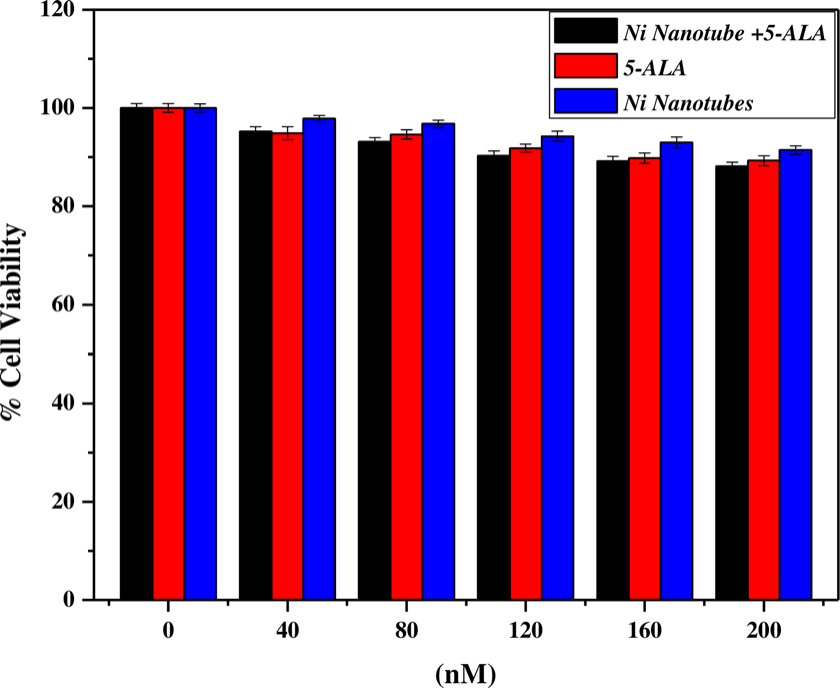
% Cell Viability of Ni Nanotubes and their complex 5-ALA. It shows that the polydisperse nature of Ni NTs has a profound effect on drug loading efficiency.

Fig 5 shows that the polydisperse nature of Ni NTs has a profound effect on drug loading efficiency. Uptake of 5-ALA individually and complexed with Ni NTs after 20 hours of incubation time was investigated. Uptake was determined for the different treatment arms of Ni NTs, 5-ALA and Ni NTs+5-ALA in HeLa cell culture plates, which were incubated with selected concentrations to have clear measurements of cytotoxicity and %cell viability loss. As the concentration increases from 40 nM to 120 nM, a marginal loss in %cell viability was observed, while for 160 nM to 200 nM, a significant loss was observed. When HeLa cells were incubated with Ni NTs alone, some variations in %cell viability loss were observed. However, the %loss of cell viability with 5-ALA alone is more prominent compared to that with Ni NTs alone. A negligible cytotoxic effect of Ni NTs has proven to be effective for its use as a drug delivery agent in different studies. However, when Ni-NTs were photosensitized with drugs and incubated for 20 hours, the complex showed a greater loss in malignant cells that increased gradually with the increase in concentration and reached a maximum at 200 nM.

This demonstrated that, at this concentration of drug, i.e., 5-ALA and Ni NTs, PDT was highly effective. In addition, we observed that different concentrations of 5-ALA alone, Ni NTs alone and Ni NTs+5-ALA have different toxic effects. It also showed the positive and satisfactory concentration dependence of cytotoxicity in HeLa cells as well as the response of PDT with Ni NTs+5-ALA. We are of the opinion that either Ni NTs are not entering the targeted site or Ni NTs are biocompatible (having no toxic effects towards HeLa cell line). The reasoning behind this is that the drug enters without internalization of Ni NTs carriers. At higher concentrations, there is significant reduction in %cell viability after treatment with 5-ALA and their combination with Ni NTs in the cervical cellular model. We investigated the possibility that a reduction in cell viability is time- and concentration-dependent, and we aimed to increase the percentage of cell apoptosis [19–21, 24].

**Fig 6:**
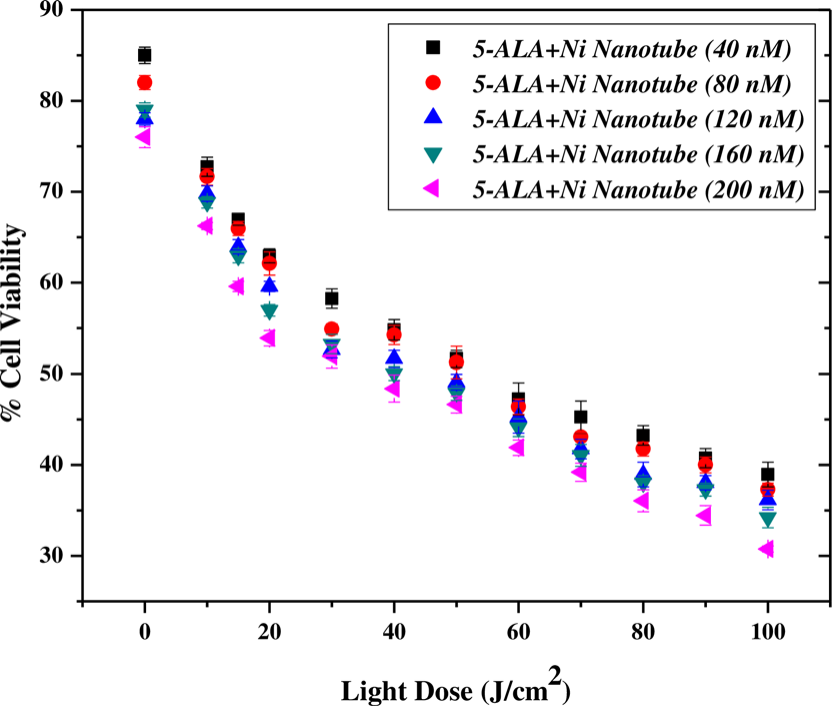
%Cell Viability of Nano-photosensitizer complex under Laser Irradiation. It shows the compatibility of 200 nM of the complex of Ni NTs with 5-ALA under laser irradiation of 20–80 J/cm^2^ for cervical cancer treatment.

Fig 6 shows the compatibility of 200 nM of the complex of Ni NTs with 5-ALA under laser irradiation of 20–80 J/cm^2^ for cervical cancer treatment. It is observed that the optimal concentration of Ni NTs+5-ALA has a promising potential for further research as a clinical treatment selective for cervical cancer in light doses. In the present experimental scheme, different concentrations of Ni NTs along with 5-ALA (having an equal v/v ratio) of 40 nM, 80 nM, 120 nM, 160 nM and 200 nM were tested for the presence of reactive oxygen species (ROS) under a suitable laser dose (≈100 J/cm^2^).

**Fig 7:**
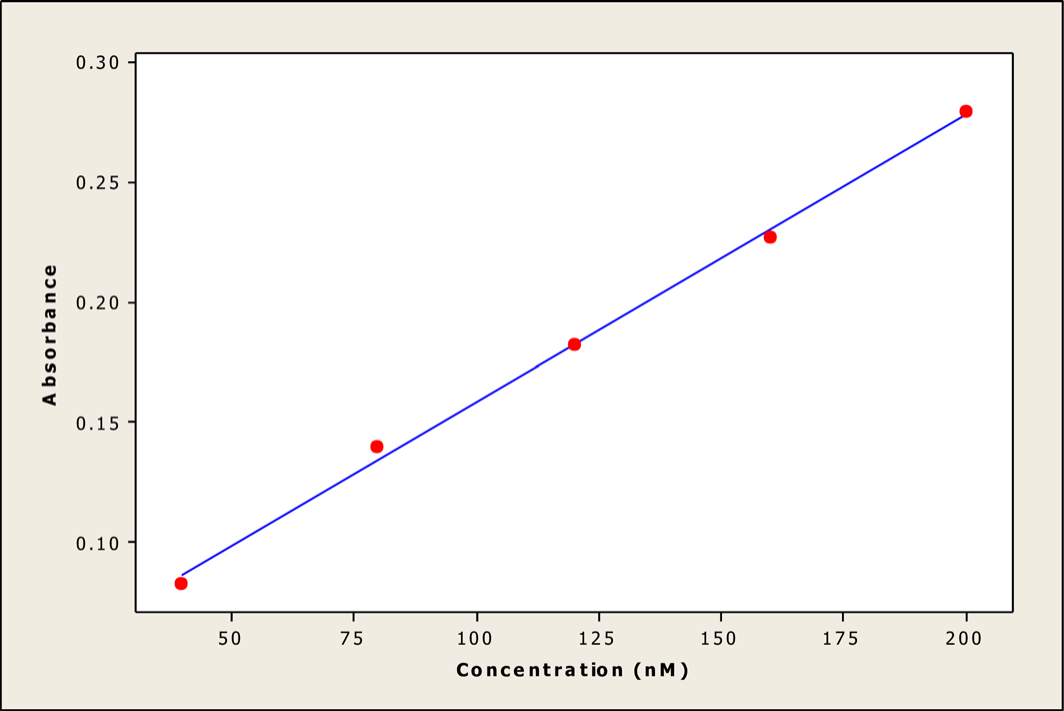
Linear calibration plot of nickel nanotubes (Ni NTs) (Conc. 40–200 nM). A graph of linear calibration was plotted for nickel nanotubes (Ni NTs) (concentration: 40–200 nM), demonstrating linearity and regression data. The linearity represents the ability of the regression equation analysis (Y = 0.0377 + 0.00120 X) from the calibration data attained (n = 5) using Ni NT absorbance vs. concentration.

**Fig 8:**
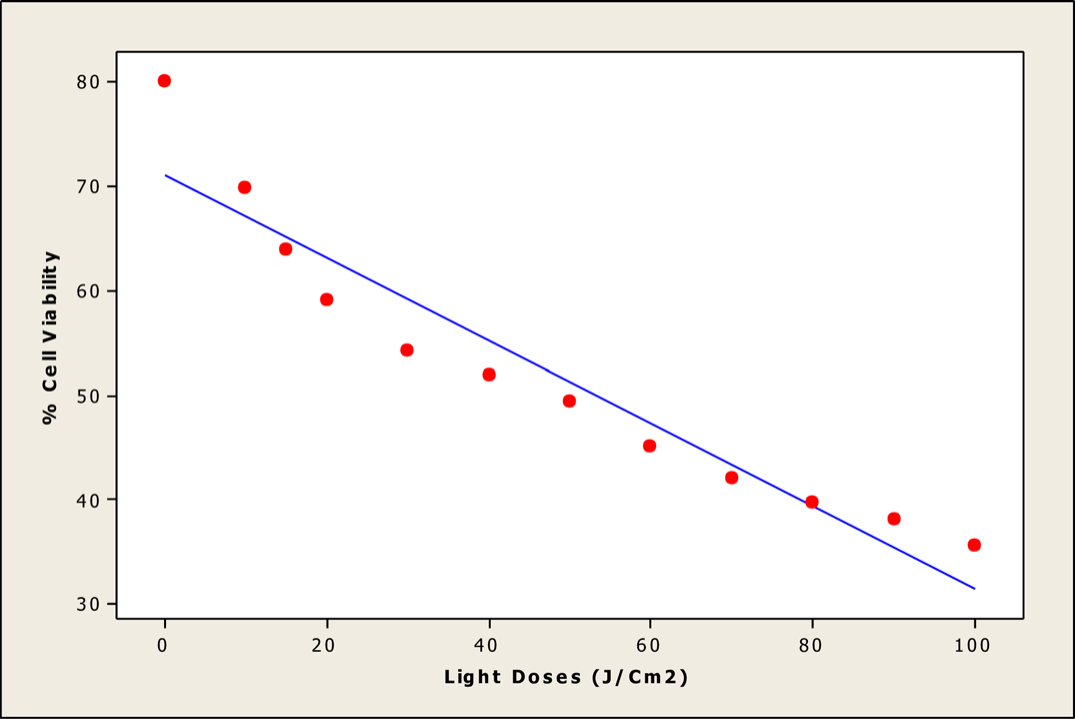
Linear calibration plot of nickel nanotubes (Ni NTs) (Light Doses (20–100 J/cm^2^). In this figure, % cell viability after treatment with Ni NTs was plotted against light doses of 20–100 J/cm^2^, showing linear calibration and a linearity using regression equation analysis (Y = 71–0.396 X) of the calibration data (n = 5).

The parameters used in this statistical analysis focused on a simple linear regression equation, which provides important information, such as coefficient determination, intercept, slope, standard deviation and variance of the slope of the regression line. To evaluate the performance parameters of the statistical analysis, a mean (X) was defined from five independent determinations, such as the concentration of Ni NTs, for the available data. Different statistical parameters were calculated to validate the experimental results. A graph (Fig 7) of linear calibration was plotted for nickel nanotubes (Ni NTs) (concentration: 40–200 nM), demonstrating linearity and regression data. The linearity represents the ability of the regression equation analysis (Y = 0.0377 + 0.00120 X) from the calibration data attained (n = 5) using Ni NT absorbance vs. concentration. The intercept has a value of 0.0377 with a slope 0.00120, and the variance of the slope of the regression line (S_o_^2^ = 4.3 x 10−^9^) is significantly acceptable. The other parameters include standard deviation (SD = 6.54 x 10^−5^), correlation coefficient (r^2^ = 0.998), and t_sa_ (8.70) and t_sb (_36.76), with p value = 0.003 where t_sa_ and t_sb_ are confidence limits for intercepts and slope. In Fig 8, %cell viability after treatment with Ni NTs was plotted against light doses of 20–100 J/cm^2^, showing linear calibration and a linearity using regression equation analysis (Y = 71–0.396 X) of the calibration data (n = 5). The intercept has a value of 71 with a slope of -0.396, and the variance of the slope of regression line (S_o_^2^ = 7.3 x 10−^3^) is significantly acceptable. The other parameters (intercept) include standard deviation (SD = 8.52 x 10^−2^), correlation coefficient (r^2^ = 0.915), and t_sa_ = 32.81 and t_sb =_ -10.38, with p = 0.000. Statistical results corroborate the accuracy of the experimental data.

**Fig 9:**
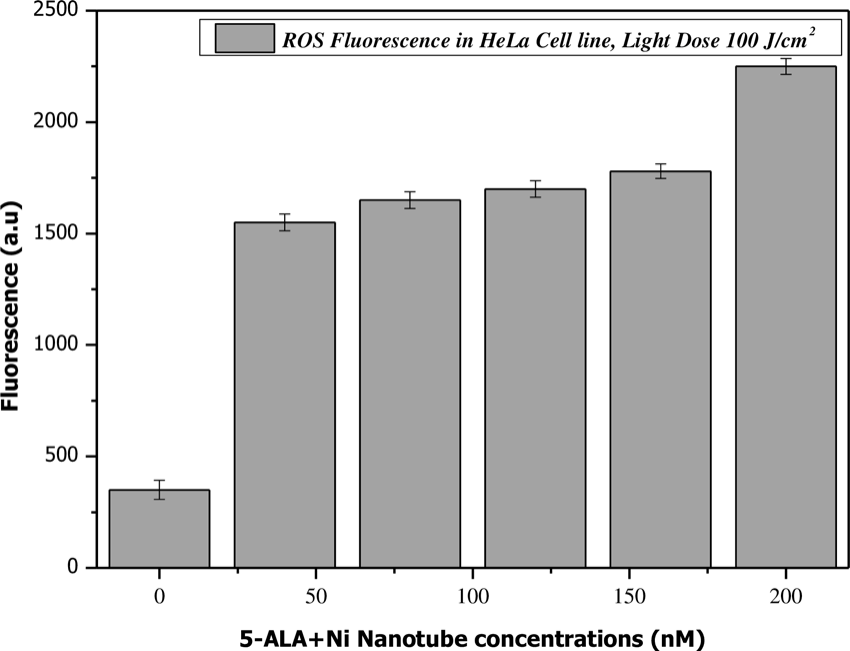
Detection of ROS Production in Ni NTs exposed HeLa cells. As is clearly observed in this figure, a homogenous rise in fluorescence of ROS production was observed, which depended on the concentration of Ni NTs +5-ALA in HeLa cell line.

As is clearly observed in Fig 9, a homogenous rise in fluorescence of ROS production was observed, which depended on the concentration of Ni NTs +5-ALA in HeLa cell line. The converse is the case in a controlled HeLa cell model. In the absence of Ni NTs +5-ALA, very nominal fluorescence was observed, which is intrinsic normal fluorescence of natural chromospheres due to metabolic activity of cervical cells. At 200 nM of Ni NTs +5-ALA, extreme levels of ROS fluorescence were revealed, which is in good agreement with the loss in cell viability. A resemblance to previously reported data was observed in the current experimental analysis [16, 17]. In this study, significant loss in cell viability was confirmed by applying apoptotic analysis strategies e.g., NRA analysis and ROS detection, in the absence and presence of laser dose. An overall trend for loss in cell viability was recorded for different concentrations of 80 nM-200 nM of the complex of Ni NTs+5-ALA under a range of laser irradiation parameters, which was approximately the same. Md. Zakir Hossain et al. explored data to find a reason for the apoptotic effect of Ni NWs in human pancreatic adenocarcinoma cells [26]. They concluded that, even individually, some concentrations of Ni NWs show significant toxicity for their biological model. In another study, it was demonstrated that Ni NWs induced HeLa cell damage via the generation of ROS and the induction of apoptosis. In our study, when 200 nM of Ni NTs+5-ALA in the presence of 100 J/cm^2^ laser dose was selected, loss in cervical cell viability was 65%, which is very significant.

The current study shows that Ni NTs complexed with 5-ALA under suitable light exposure not only arrest the cell cycle but also inhibit HeLa cell proliferation by inducing oxidative stress through the liberation of reactive oxygen species (ROS). More specifically, it was found through experimental examination that individual Ni NTs cannot trigger significant apoptosis in our cellular model; furthermore, it causes less toxicity than the Ni NTs+5-ALA.

## 4. Conclusion

This study concludes that loss in HeLa cell viability reaches 65% with an effective dose of 200 nM of Ni NTs+5-ALA when irradiated with 100 J/cm^2^ of a diode laser (630 nm wavelength). In addition, an ongoing experimental study has verified that Ni NTs are capable of inducing dose- and exposure time-dependent cytotoxicity in a cervical cancer model. These data revealed that the toxicity of Ni NTs in the HeLa cell line is dose dependent. Furthermore, chemical activity as well as shape and size of Ni NTs can cause liberation of ROS, which can cause vascular shutdown in a cervical cell model. Ni NTs exhibit multiple anti-proliferative effects, including cell growth inhibition, ROS generation, cell cycle arrest, and cell apoptosis. Further careful study may lead to more refinement, which may also lead to greater efficiency and enhancement of treatment parameters in local as well as malignant treatment. To potentially renew and aid previous drug sensitivity evaluation studies, a statistical analysis based on linear regression was performed on the experimental results to help to understand the mechanism of drug action on the tumor cells.

## Supporting Information

S1 Fig Linear calibration plot of nickel nanotubes (Ni NTs) (Conc. 40–200 nM). A graph (Fig 7) of linear calibration was plotted for nickel nanotubes (Ni NTs) (concentration: 40–200 nM), demonstrating linearity and regression data. The linearity represents the ability of the regression equation analysis (Y = 0.0377 + 0.00120 X) from the calibration data attained (n = 5) using Ni NT absorbance vs. concentration.
(TIF)

S2 Fig Linear calibration plot of nickel nanotubes (Ni NTs) (Light Doses (20–100 J/cm^2^). In this figure, % cell viability after treatment with Ni NTs was plotted against light doses of 20–100 J/cm^2^, showing linear calibration and a linearity using regression equation analysis (Y = 71–0.396 X) of the calibration data (n = 5).
(TIF)
