# Supplementary figures and images for: Photodynamic Effect of Ni Nanotubes on an HeLa Cell Line
Source: PLoS One. 2016 Mar 18;11(3):e0150295. doi: 10.1371/journal.pone.0150295 (PMC4798439; doi:10.1371/journal.pone.0150295)

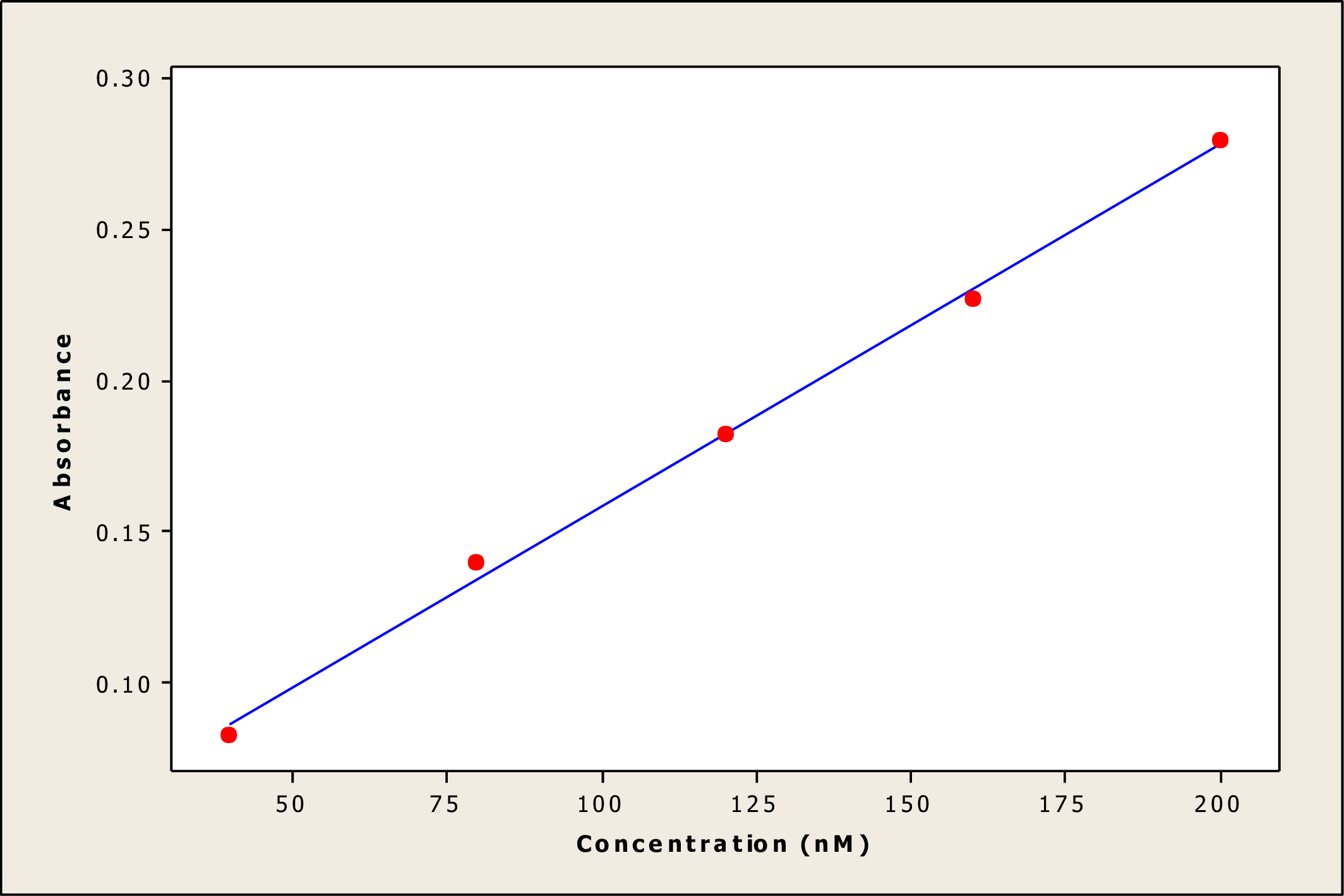

Supplement: S1 Fig — A graph (Fig 7) of linear calibration was plotted for nickel nanotubes (Ni NTs) (concentration: 40–200 nM), demonstrating linearity and regression data. The linearity represents the ability of the regression equation analysis (Y = 0.0377 + 0.00120 X) from the calibration data attained (n = 5) using Ni NT absorbance vs. concentration. (TIF) [file pone.0150295.s001.tif]

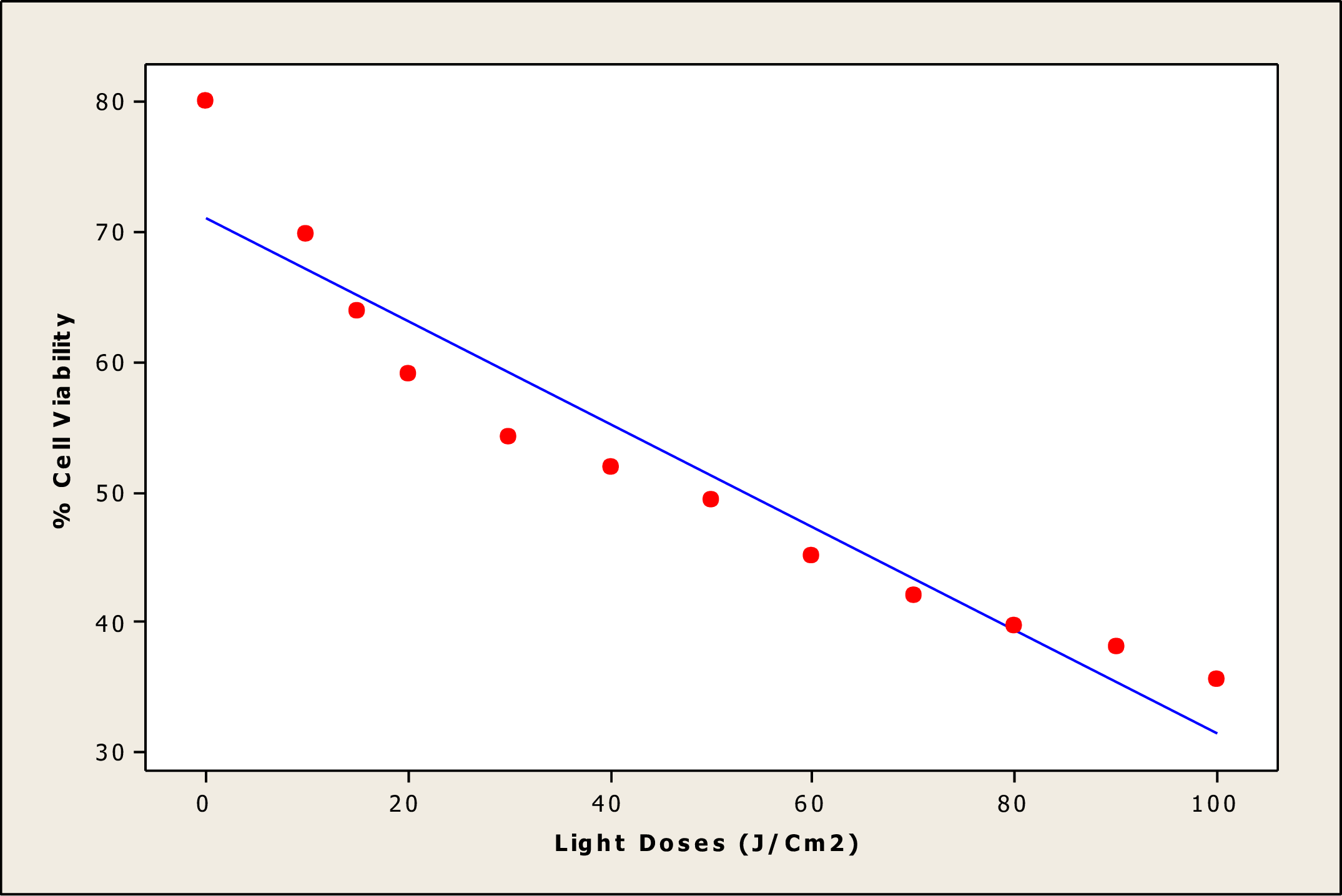

Supplement: S2 Fig — In this figure, % cell viability after treatment with Ni NTs was plotted against light doses of 20–100 J/cm2, showing linear calibration and a linearity using regression equation analysis (Y = 71–0.396 X) of the calibration data (n = 5). (TIF) [file pone.0150295.s002.tif]
